# The Effect of Fluorescent Light on Anxiety Patients

**DOI:** 10.7759/cureus.13436

**Published:** 2021-02-19

**Authors:** Raghad F Khorshid, Sakhaa H Almadani, Amjad M Al shehri, Lama M Abduljawad, Ahmad M Alsaleh

**Affiliations:** 1 College of Medicine, King Saud Bin Abdulaziz University for Health Sciences, Jeddah, SAU; 2 Psychiatry, King Abdulaziz Medical City, Jeddah, SAU

**Keywords:** fluorescent light, mood, anxiety, anxiety disorders, psychiatry

## Abstract

Aim

Anxiety is an emotion recognized by a feeling of tension and agitation along with physiological excitement. Several factors could influence the moods, emotions, and behaviors of patients with anxiety disorders. One of these factors includes exposure to different lightings. In lots of working environments, fluorescent lights are the most dominant light source. Due to the dominance and exposure of fluorescent light, it has been proven that it could have different effects on the human body. Up to our knowledge, no previous or recent studies addressed the relationship between fluorescent light and anxiety disorders, even though based on observations, many patients with anxiety disorders have complained when exposed to fluorescent light. This research determined whether fluorescent light caused discomfort and amplified anxiety symptoms in anxiety patients in comparison to healthy individuals. In other words, the purpose is to determine the effect of fluorescent light on anxiety patients.

Methods

The study design was comparative cross-sectional. Two questionnaires were used, one was a validated screening tool called the Mini-International Neuropsychiatric Interview (MINI), which was used to screen participants for mental disorders. The second was a self-administered, piloted, and validated questionnaire that included questions regarding the effects of fluorescent light on participants. This study was carried out in the outpatient clinics of King Abdulaziz Medical City in Jeddah between July 2019 and November 2019. A non-probability consecutive sampling technique was used.

Results

The sample size was 206 participants. Seventy-five percent of participants with anxiety disorders agreed that they do not feel comfortable with the lighting of this clinic more than healthy participants that were only 25.0% (P = 0.007). When exposed to a room with fluorescent lighting, most of the participants with anxiety disorders would try to adapt to the lights (66.7%) or leave the room (73.7%) than healthy participants (P = 0.007). Furthermore, fluorescent light reminded participants of anxiety disorders of “old house and old places,” “headaches, negativity, and discomfort,” and “hospitals and schools.”

Conclusion

Participants with anxiety disorders are affected by fluorescent light. They feel uncomfortable and would prefer to either leave the place with fluorescent light or try to adapt. Fluorescent light reminds anxiety participants of negative aspects more than healthy participants.

## Introduction

Anxiety is an emotion recognized by a feeling of tension and agitation along with physiological excitement [1]. According to the fifth edition of the Diagnostic and Statistical Manual of Mental Disorders (DSM-5), there are several types of anxiety disorders including generalized anxiety disorder, social anxiety disorder, specific phobia, panic disorder, and agoraphobia [2]. Generalized anxiety disorder is the excessive and uncontrollable worry about different activities or events that persists for several days for at least six months. Social anxiety disorder, or social phobia, is the excessive fear of social situations that involve interacting with others. Specific phobia is characterized by an extreme, persistent, and irrational fear of a specific object or situation [3]. Social anxiety disorder and specific phobia are the most common types of anxiety disorders [4]. Panic disorder is the sudden onset of an unexpected and recurrent attack of anxiety, which is accompanied by a feeling of being out of control or dying. Agoraphobia is the fear of public places that are considered difficult to escape from or find help [3]. Regarding the gender differences, in general, females have approximately twice the risk of developing anxiety disorders more than males [2]. A systemic review and a meta-regression stated that the global prevalence of anxiety disorders ranges from 0.9% to 28.3% [5]. Anxiety disorders appear to share a wide range of risk factors involving stressful life events and family history of anxiety disorders [6]. Additionally, it has been found that there are other factors associated with the high incidence of anxiety disorders and these include female gender, middle age, and poor education [7]. Due to the abundance of risk factors, identifying other risk factors could help in prevention, early intervention, and controlling these disorders. 

Several factors could impact our mood, emotions, and behaviors. One of these factors includes exposure to different lightings [8]. In lots of working environments, fluorescent lights are the most dominant light source. A fluorescent light lamp is filled with gases such as mercury vapor and is coated with phosphor on the inside. When electricity flows through the vapor, ultraviolet light is produced. The phosphor then absorbs the ultraviolet light and releases visible light [9]. The reason behind the dominance of fluorescent light in offices, hospitals, schools, and most working environments is the belief that fluorescent light is similar to natural daylight [10]. Fluorescent light is also used in electronics such as smartphones, television, and computers [11]. Due to the dominance and exposure of fluorescent light, it has been proven that it could have different effects on the human body. The human eye contains proteins that are sensitive to light and are called melanopsins [12]. These proteins are responsible for detecting the intensity of light and thus could affect the body. Moreover, the effects are directly proportional to the lighting’s intensity. A type of light that has high intensity is fluorescent light, which could increase the effects on individuals and therefore lead to different responses [12]. A study done in Helsinki, Finland in 2002 showed that fluorescent light, mentioned in the study title as "bright light," increases alertness, but has a negative outcome on sleep. This increase of alertness is stated to have a positive impact because it improves cognitive performances and mood when exercising [8]. However, fluorescent light exposure can have negative effects when intending to relax, and it could disturb the sleeping cycle and the circadian rhythm (the body’s biological clock), could be due to the decrease in melatonin (a hormone that regulates the circadian rhythm) [13-15]. The effect of lighting also differs with gender. A previous study showed that females had more negative moods and lower problem-solving scores in cold light (white light) in comparison to warm light (yellow light), whereas, for males, the effects were the opposite [16]. In other situations, it has been found that high-intensity light increases the visual performance which makes it a more comfortable study environment; however, the speed of reading from devices emitting fluorescent light is less than reading from printed text due to the negative effect on the ocular motion [17,18]. Another study also found that the exposure to dark light at work had a negative effect on the mood of workers, and exposure to brighter light had a positive effect, but then the moods declined as the light became excessively bright [19,20].

Several articles have discussed the effects of lighting on mood, cognition, and sleep; however, up to our knowledge, no previous or recent studies addressed the relationship between fluorescent light and anxiety disorders, even though based on observations, many patients with anxiety disorders have complained when exposed to fluorescent light [8,13-20]. Since fluorescent light is used worldwide, it would be beneficial to know whether it would impact patients with anxiety disorders. There was no specific type/brand of fluorescent light used in previous studies, each research used a different type. 

The purpose of this study was to determine whether fluorescent light affected anxiety patients. This research also considered other factors that might affect the answers of the participants such as age, gender, and educational level. Furthermore, this study compared different anxiety disorders with the fluorescent light effect. This study could increase the knowledge and awareness about the impact of the environment on patients with anxiety disorders, and it could help in adjusting the type of lighting when needed in certain settings as hospitals and waiting areas.

## Materials and methods

This study was approved by the Institutional Review Board committee of King Abdullah International Medical Research Center. The study design is a comparative cross-sectional study, and it was conducted in the outpatient clinics of KAMC in Jeddah, Saudi Arabia. The clinics consisted of a 4x4m room, windows covered with curtains, one bed for the patient, fluorescent lights, one computer, two chairs, and plain white walls. Distractions such as noise and unneeded materials were eliminated as much as possible in order to not interfere with the study results. The type of lighting used in the clinic is the Osram Lumilux cool daylight 28W/865 fluorescent light.

Inclusion and exclusion criteria of the participants

All the participants with any type of anxiety disorders that agreed to participate were included after obtaining their consent. Also, the participants were over the age of 18 and under the age of 65 because the study targeted adults only. All participants were Saudis, as the majority of patients treated in this health care facility were Saudis. Furthermore, all participants were living in the western region. The inclusion criteria of the control group were identical to the cases, participants with anxiety disorders, but with no mental illnesses. The participants with endocrine disease-induced anxiety, or even medical illness-induced or anxiety due to medical illnesses such as Graves' disease, hyperthyroidism, and pheochromocytoma were excluded.

Sample size and sampling technique

The sample size was calculated from an authorized online software (https://stat.uiowa.edu). The predicted proportion of healthy individuals that are affected by fluorescent light is 30%, and the predicted proportion of patients with anxiety disorders is 80%. A total sample size of 206 is calculated with an alpha of 0.5 and a beta of 80% (103 healthy individuals and 103 patients with anxiety disorders).

The sampling technique used was a non-probability consecutive sampling because not all the population has the same chance of being selected, only all the patients that present to the clinic.

Data collection process

The data were collected in the outpatient clinics of King Abdulaziz Medical City in Jeddah between July 2019 and November 2019. A comparative cross-sectional study was used and two questionnaires were required. The first questionnaire is called the “Mini-International Neuropsychiatric Interview” that screens for all mental illnesses. This questionnaire’s purpose is to confirm participants’ diagnoses, screen for any other possible mental illness, and ensure that the healthy participants have no mental illnesses. 

The second questionnaire is a self-administered questionnaire designed by the investigators and was revised and validated by the primary investigator (PI), a consultant psychiatrist. This questionnaire covers the demographics such as age, gender, nationality, city of residence, marital status, and educational level. It also includes questions related to the presence of any chronic illness, and if they were diagnosed with any anxiety disorder (Appendices 1-4). If the study subject was diagnosed with an anxiety disorder before, he/she was asked more questions related to the type of anxiety disorder and the time of the diagnosis, and if they received therapy or medications. After the demographics, the participants were exposed to fluorescent lights for around 10-15 minutes and meanwhile were asked questions related to their thoughts and feelings about the lights. The questions concerning the lighting were five questions. Three were multiple-choice, and the other two were open-ended questions to allow the participants to answer without restrictions. The questionnaire’s reliability was assessed using a pilot study on 50 participants and the Cronbach alpha was measured. The Cronbach alpha value was 0.7.

Data analysis

The data collected were entered in excel and analyzed by Statistical Package for the Social Sciences (SPSS) version 23 (IBM Corp, Armonk, NY). As for the inferential statistics, the chi-square test was used to compare between two proportions, and a one-way analysis of variance (ANOVA) test was used to compare between more than two proportions. The open-ended questions were represented in the description.

## Results

As for the demographics and characteristics of the participants, approximately equal numbers of males and females participated in this study. A total of 206 subjects were recruited, of which four were excluded early on because of missing data, making a final sample size of 202 subjects that were included (97 cases and 105 healthy controls). There was no difference in gender between the two groups. The average age of participants was 34 years old. Most of the participants were from Jeddah, and most participants’ education levels were at the university or college level. As for the social status, the majority of the participants were married (Table 1).

**Table 1:** Comparison between cases and control in relation to demographics (N = 202).

Demographics	N (% or mean ± SD) (n=202)
Gender	
Male	99 (49.0%)
Female	103 (51.0%)
Age	33.6 ± 12.6
City of residence	
Jeddah	139 (68.8%)
Makkah	38 (18.8%)
Al-Madinah	4 (2.0%)
Others	21 (10.4%)
Education level	
Up to primary or middle school	12 (5.9%)
Up to high school	59 (29.2%)
University/college	106 (52.5%)
Postgraduate	14 (6.9%)
Diploma	11 (5.4%)
Marital status	
Single	80 (39.6%)
Married	112 (55.4%)
Divorced	10 (5.0%)

All the participants were asked a total of six items/questions, excluding the demographics and other participant information, that were lighting-related (Appendix 4). Three of those questions were combined in one table. The responses were divided into two groups, healthy participants and participants with at least one anxiety disorder. As for the preference of lighting, more healthy, 60 (58.8%) of participants preferred fluorescent over incandescent in comparison to 42 (41.2%) participants with anxiety disorders that choose fluorescent lighting. On the other hand, 35 (62.5%) of participants with anxiety disorders preferred incandescent over fluorescent in comparison to 21 (37.5%) healthy participants that chose the incandescent light option. However, the difference is not significant (P = 0.079). Participants that did not choose either option, fluorescent or incandescent, chose both as their preference or chose a different light color as red light. A significantly higher number of participants with anxiety disorders agreed with the statement that says “I do not feel comfortable with the lighting of this clinic” compared to healthy participants (75.0% vs 25.0%) (P = 0.007). Regarding the last variable, participants were asked how they would react if they were in a room that had fluorescent light, and they had to choose between three options, “try to adapt,” “leave the room,” or do nothing. More of the participants with anxiety would try to adapt or leave the room than healthy participants. As for participants with anxiety disorders, 66.7% would try to adapt to the lights, and 73.7% would leave the room versus 33.3% and 26% of healthy individuals, respectively. The participants with anxiety disorders (42.6%) would not do anything at all, while healthy participants (57.4%) would do the same (P = 0.007; Table 2).

**Table 2:** Shows the association between the participants with at least one anxiety disorder and the effect of fluorescent light. N = 202 participants and the frequencies of the associations were added as well. P < 0.05 is significant.

Questionnaire questions	Has at least one anxiety disorder (n=97)	Does not have an anxiety disorder (n=105)	P-value
Which type of light do you prefer?			0.079
Fluorescent	42 (41.2%)	60 (58.8%)	
Incandescent	35 (62.5%)	21 (37.5%)	
Both	17 (44.7%)	21 (55.3%)	
Others	3 (50.0%)	3 (50.0%)	
I do not feel comfortable with the lighting of this clinic			0.007
Agree	21 (75.0%)	7 (25.0%)	
Disagree	73 (44.2%)	92 (55.8%)	
I don’t know	3 (33.3%)	6 (66.7%)	
What would you do if you were in a room that had fluorescent light?			0.007
Try to adapt to the lights	14 (66.7%)	7 (33.3%)	
Leave the room	14 (73.7%)	5 (26.3%)	
Nothing	65 (42.6%)	93 (57.4%)	

**Figure 1:**
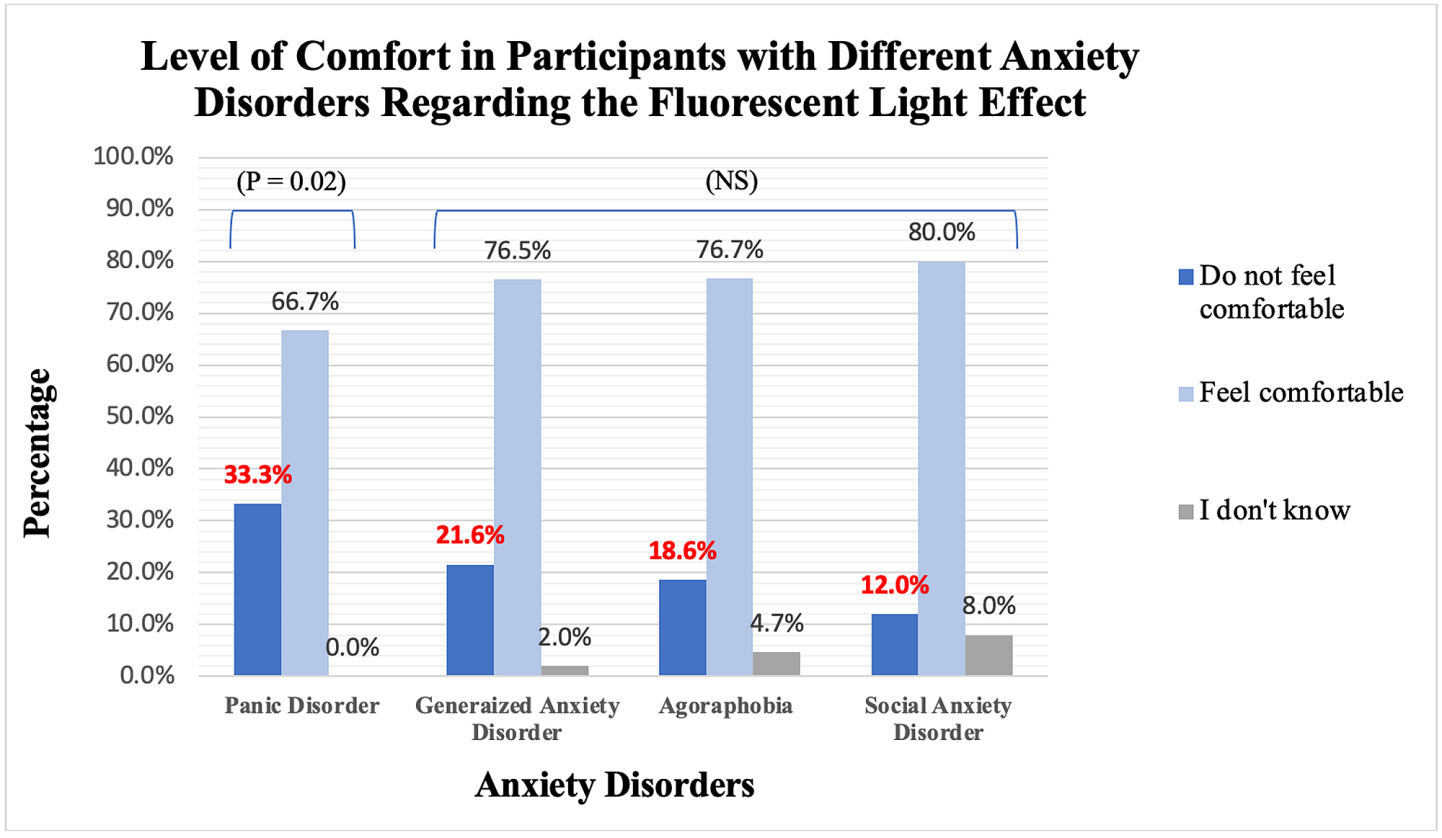
A bar chart representing the association between the participants with different anxiety disorders and the statement “I do not feel comfortable in the lighting of this clinic.” N = 202 participants and the frequencies of the associations were added as well. P < 0.05 is significant. NS = not significant

Figure 1 shows the association between different anxiety disorders and the level of comfort to fluorescent light. The results showed a non-significant difference in people with generalized anxiety disorder, agoraphobia, or social anxiety disorder. However, participants with panic disorders had a significantly lower level of comfort with fluorescent light by their agreement with the statement “I do not feel comfortable with the lighting of this clinic” (P = 0.02; Figure 1).

Other factors such as the participants' gender, age, and educational level were examined for their effect on fluorescent preference. The majority of males, 56 (56.6%) prefer fluorescent light, while females prefer both fluorescent 46 (44.7%) and incandescent 44 (42.7%) approximately equally (P = 0.00). As for the age of the participants, most participants that chose incandescent were approximately 7 years younger than participants that chose fluorescent (P = 0.00), which was significant. As for the educational level, the difference in preference was insignificant (Table 3).

**Table 3:** This table shows the association between the participants’ demographics and the lighting preference (N = 202). P < 0.05 is significant.

Demographics	What type of lighting do you prefer?				P-value
	Fluorescent	Incandescent	Both	Others	
Gender					0.000
Male	56 (56.6%)	12 (12.1%)	27 (27.3%)	4 (4.0%)	
Female	46 (44.7%)	44 (42.7%)	11 (10.7%)	2 (1.9%)	
Age	36.1 ± 12.2	29.52 ± 10.6	30.7 ± 12.2	47.3 ± 20.4	0.000
Level of education					0.102
Up to primary or middle school	8 (7.8%)	1 (1.8%)	1 (2.6%)	2 (33.3%)	
Up to high school	31 (30.4%)	14 (25.0%)	12 (31.6%)	2 (33.3%)	
University/college	49 (48.0%)	37 (66.1%)	19 (50.0%)	1 (16.7%)	
Post Graduate level	7 (6.9%)	3 (5.4%)	3 (7.9%)	1 (16.7%)	
Diploma	7 (6.9%)	1 (1.8%)	3 (7.9%)	0 (0.0%)	

Three questions from the six items in the questionnaire were qualitative (Appendix 4). The participant had the freedom to write whatever they prefer. The most important question asked was “what does fluorescent light remind you of?” Most of the participants did not write an answer, or they wrote “nothing,” while the rest of the participants had answered the question. As for the participants who had at least one anxiety disorder, some answers were repetitive. Fluorescent light reminds them mostly of “old house and old places,” “headaches, negativity, and overwhelm,” and “hospitals and schools”

On the other hand, there were also common responses among healthy participants. Several participants stated that fluorescent light reminds them of “happiness and comfort” and “brightness and mornings.” Therefore, it can be deduced that several anxiety participants had a more negative response, while healthy individuals had positive responses. The second question that was asked was “what do you dislike about this clinic?” The participants had the freedom to choose whatever element of the clinic that they disliked. It did not necessarily have to be related to the lighting. There were some common responses among participants with anxiety disorders. They disliked the “lighting” and the “small clinics.” As for the participants that do not has anxiety disorders, they disliked the “lighting,” the “waiting time,” and the “noise.” Several participants chose ‘lighting’ as the element that they dislike, but more participants that chose this option had anxiety disorders.

## Discussion

In this study, more participants with anxiety disorders did not feel comfortable with the lighting of the clinic, would try to adapt to the lights, and would leave the room more than healthy participants.

Various studies have stated that full-spectrum fluorescent lighting could improve cognitive performance, vision, and mood [10]. A recent study that discussed digital media and sleeps in childhood and adolescence concluded that there is an adverse association between screen-based media consumption and sleep health. One of the mechanisms behind that conclusion was the effects of light emitted from devices on circadian timing, sleep physiology, and alertness [11]. This study and several other studies showed that fluorescent light increases alertness but has a negative outcome on sleep and relaxation [13]. In our study, since it focuses on anxiety, participants with anxiety disorders are already alert when in a state of anxiety, and so additional alertness from fluorescent light may occur. This also correlates with the idea that anxiety does not allow relaxation in addition to the fluorescent light that decreases relaxation. In terms of mood changes, a study concluded that fluorescent light not only increases alertness but also positively impacts mood in healthy participants [21]. This correlates with our study, which showed that healthy participants had more positive responses regarding fluorescent light. Many stated that fluorescent light makes them feel happiness and comfort. 

A previous study concluded that bright light suppresses melatonin and increases body temperature, therefore, it makes the subjects stay awake for longer durations and kept them alert with better performance. However, this exposure led the subjects to experience mood deterioration and loss of motivation the next day [17]. In regard to our study, the findings showed that fluorescent light exposure is not preferred in anxiety patients, but we did not specify the duration of exposure. Another study suggests that fluorescent light is effective in decreasing depressive symptoms and alleviating mood disorders [22], while our study shows that fluorescent light might negatively affect anxiety patients and make them feel uncomfortable. This shows that the effect of fluorescent light could vary depending on the disorder. 

After completing the research, some limitations have been recognized. The first limitation was that several participants had to be excluded due to the fact that they fulfilled the exclusion criteria such as ages under 18 and non-Saudis. The second limitation was that the study only focused on one medical center, which was KAMC, and the sample size was limited, with 206 participants. The third limitation was that a few participants that we encountered were in a hurry in order to not miss their medical appointments, and that might also increase the possibility of errors. The fourth limitation was that participants with specific phobia were not included in this study since the Mini-International Neuropsychiatric Interview does not screen for specific phobia symptoms. Lastly, the exposure of patients to the fluorescent light was for a short and limited time, which was around 10-15 minutes, therefore, we did not assess the effects of fluorescent light for longer periods.

## Conclusions

Participants with anxiety disorders are affected by the fluorescent light. They feel uncomfortable and would prefer to either leave the place with fluorescent light or try to adapt to the situation. Also, fluorescent light reminds anxiety participants of negative aspects more than healthy participants. We recommend having more studies about lighting and its effect on mood and different disorders. We also recommend considering adding incandescent lighting in working places, schools, and hospitals in order to allow to maximize comfort and satisfaction for everyone. 

## Figures and Tables

**Figure 2:**
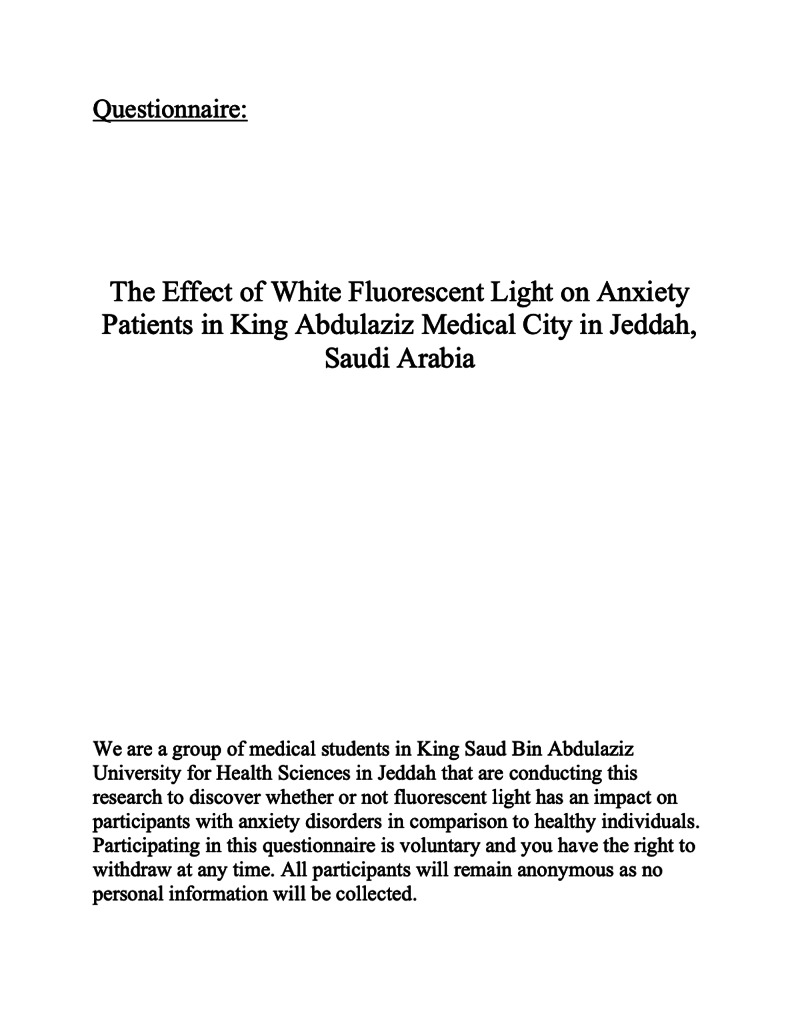
Page 1 of the self-administered questionnaire.

**Figure 3:**
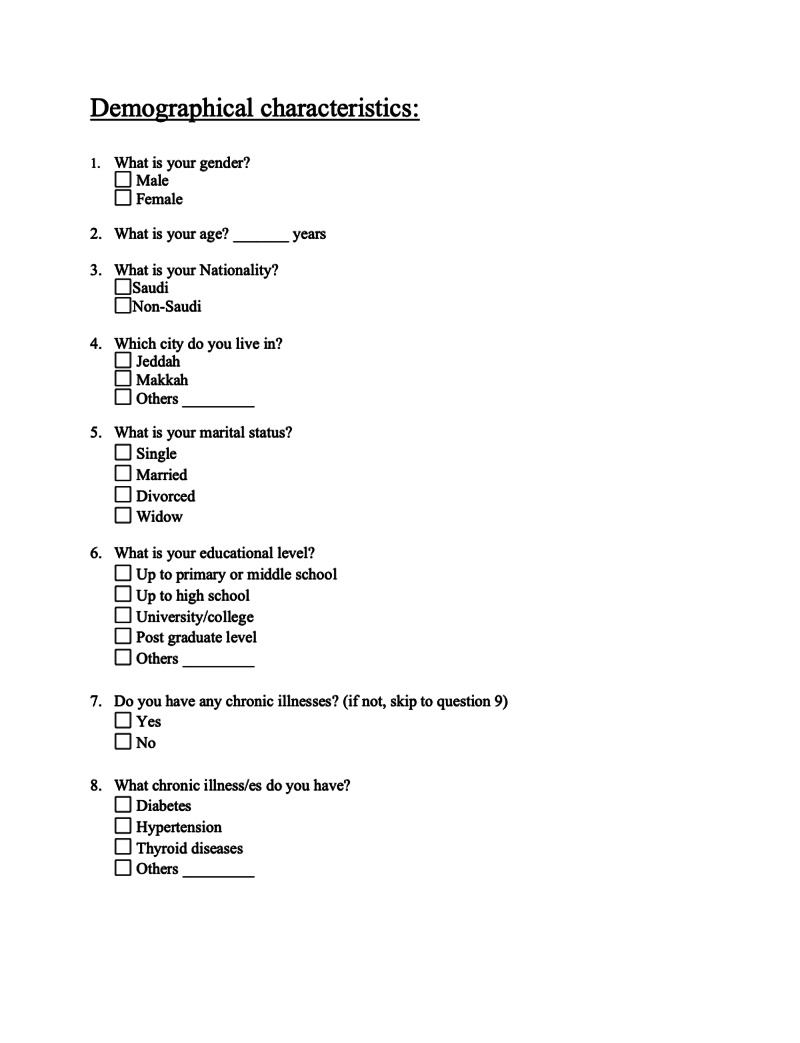
Page 2 of the self-administered questionnaire.

**Figure 4:**
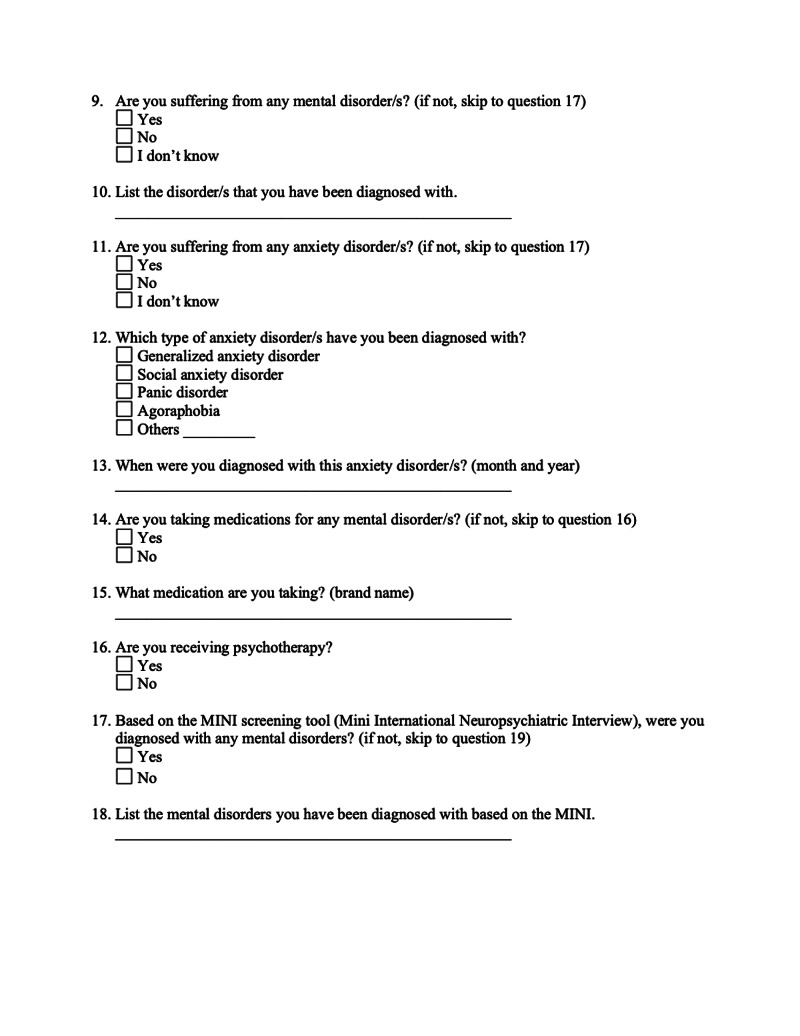
Page 3 of the self-administered questionnaire.

**Figure 5:**
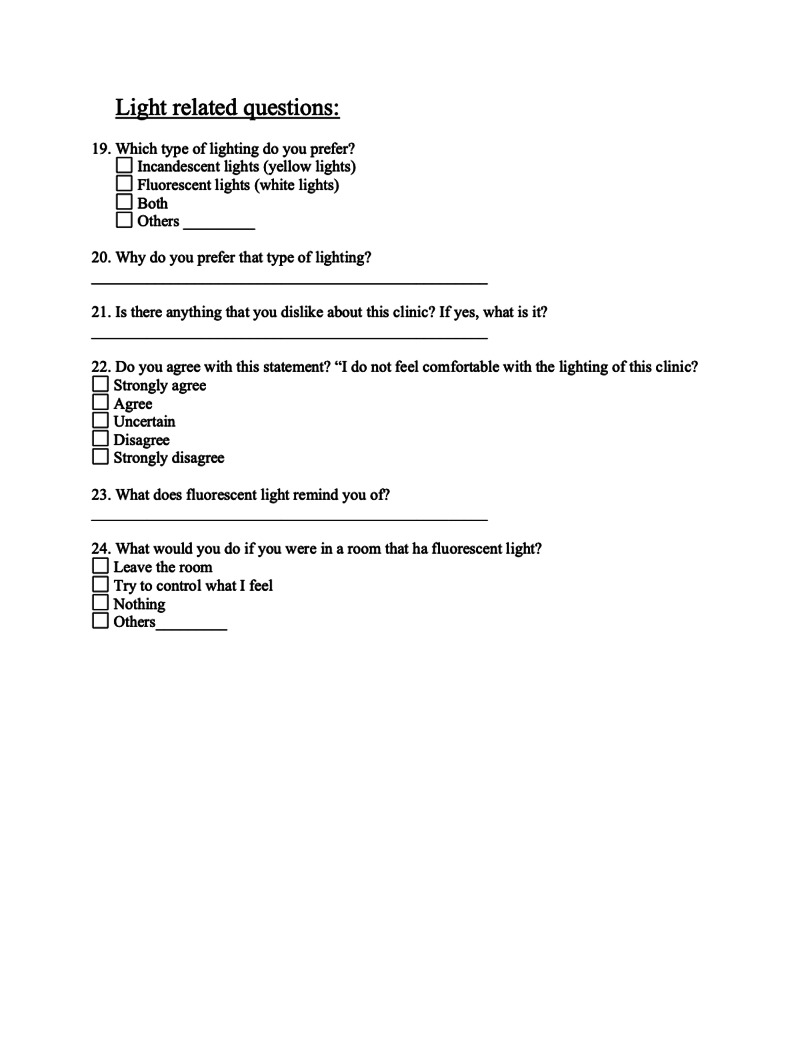
Page 4 of the self-administered questionnaire.

## Appendices

Appendix 1 

Appendix 2 

Appendix 3 

Appendix 4 
